# Update: Influenza Activity — United States, September 28, 2014–February 21, 2015

**Published:** 2015-03-06

**Authors:** World Health Organization Collaborating Center for Surveillance, Epidemiology, and Control of Influenza, Tiffany D’Mello, Lynnette Brammer, Lenee Blanton, Krista Kniss, Sophie Smith, Desiree Mustaquim, Craig Steffens, Rosaline Dhara, Jessica Cohen, Sandra S. Chaves, Lyn Finelli, Joseph Bresee, Teresa Wallis, Xiyan Xu, Anwar Isa Abd Elal, Larisa Gubareva, David Wentworth, Julie Villanueva, Jackie Katz, Daniel Jernigan

**Affiliations:** 1 Influenza Division, National Center for Immunization and Respiratory Diseases, CDC

Influenza activity in the United States began to increase in mid-November, remained elevated through February 21, 2015, and is expected to continue for several more weeks. To date, influenza A (H3N2) viruses have predominated overall. As has been observed in previous seasons during which influenza A (H3N2) viruses predominated, adults aged ≥65 years have been most severely affected. The cumulative laboratory-confirmed influenza-associated hospitalization rate among adults aged ≥65 years is the highest recorded since this type of surveillance began in 2005. This age group also accounts for the majority of deaths attributed to pneumonia and influenza. The majority of circulating influenza A (H3N2) viruses are different from the influenza A (H3N2) component of the 2014–15 Northern Hemisphere seasonal vaccines, and the predominance of these antigenically and genetically drifted viruses has resulted in reduced vaccine effectiveness (1). This report summarizes U.S. influenza activity* since September 28, 2014, and updates the previous summary (2).

## Viral Surveillance

**FIGURE 1:**
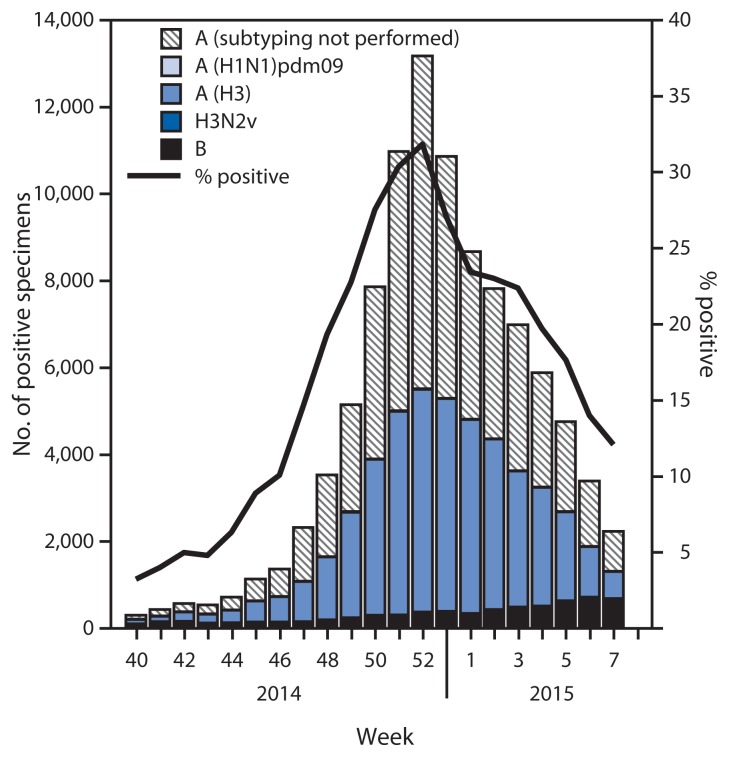
Number^*^ and percentage of respiratory specimens testing positive for influenza reported by World Health Organization and National Respiratory and Enteric Virus Surveillance System collaborating laboratories, by type, subtype, and surveillance week — United States, 2014–15 influenza season^†^ ^*^ N = 486,004. ^†^ Data reported as of February 21, 2015.

During September 28, 2014, through February 21, 2015, approximately 270 World Health Organization (WHO) and National Respiratory and Enteric Virus Surveillance System collaborating laboratories in the United States tested 486,004 respiratory specimens for influenza viruses, and 98,680 (20.3%) were positive (Figure 1). Of these, 91,837 (93.1%) were influenza A viruses, and 6,843 (6.9%) were influenza B viruses. Of the 91,837 influenza A viruses, 43,288 (47.1%) were subtyped, of which 43,123 (99.6%) were influenza A (H3) viruses and 165 (0.4%) were influenza A (H1N1)pdm09 viruses. The percentage of specimens that tested positive for influenza increased through the week ending December 27, 2014 (week 52), when 31.8% were positive and decreased subsequently. In the week ending February 21, 2015 (week 7), 12.1% of specimens tested positive. Influenza A (H3) viruses have been reported most frequently in the United States overall, followed by influenza B viruses. Influenza A (H1N1)pdm09 viruses have been rarely identified.

## Novel Influenza A Viruses

Since September 28, 2014, two human infections with novel influenza A viruses have been reported. One infection with an influenza A (H3N2) variant virus was reported to CDC during the week ending October 18, 2014 (week 42) from Wisconsin, and one infection with an influenza A (H1N1) variant virus was reported to CDC during the week ending January 24, 2015 (week 3) from Minnesota (2). The illness onsets for both patients was in October 2014. Both patients reported contact with swine in the week preceding illness, and both patients fully recovered. No further cases were identified in contacts of either patient.

## Antigenic and Genetic Characterization of Influenza Viruses

WHO collaborating laboratories in the United States are requested to submit a subset of their influenza-positive respiratory specimens to CDC for further virus characterization. CDC has antigenically and/or genetically characterized† 933 influenza viruses collected since October 1, 2014, including 27 influenza A (H1N1)pdm09, 752 influenza A (H3N2), and 154 influenza B viruses. All influenza A (H1N1)pdm09 viruses were antigenically characterized as A/California/7/2009-like, the influenza A (H1N1) component of the 2014–15 Northern Hemisphere vaccines. Of the 752 influenza A (H3N2) viruses that were characterized, 228 (30%) were characterized as A/Texas/50/2012-like, the influenza A (H3N2) component of the 2014–15 Northern Hemisphere vaccines. The remaining 524 (70%) influenza A (H3N2) viruses showed either reduced titers with antiserum produced against A/Texas/50/2012 or belonged to genetic groups that typically show reduced titers to A/Texas/50/2012. These viruses that showed reduced titers to A/Texas/50/2012 belong to multiple genetic groups and most, but not all, were antigenically similar to the influenza A (H3N2) virus selected for the 2015 Southern Hemisphere influenza vaccine (A/Switzerland/9715293/2013). A/Switzerland/9715293/2013 is related to, but antigenically and genetically distinguishable, from the A/Texas/50/2012 vaccine virus. Of the 154 influenza B viruses tested, 107 (69%) belonged to the B/Yamagata lineage. Of these, 100 (94%) were characterized as B/Massachusetts/2/2012-like, the influenza B component of the 2014–15 Northern Hemisphere trivalent and quadrivalent influenza vaccines, and seven (6%) showed reduced titers to B/Massachusetts/2/2012. The remaining 47 (31%) influenza B viruses tested belonged to the B/Victoria lineage of viruses. Of these, 43 (91%) were antigenically characterized as B/Brisbane/60/2008-like, the influenza B component of the 2014–15 Northern Hemisphere quadrivalent influenza vaccine, and four (9%) showed reduced titers to B/Brisbane/60/2008.

## Antiviral Resistance of Influenza Viruses

Since October 1, 2014, a total of 2,011 influenza viruses have been tested for resistance to influenza neuraminidase inhibitor antiviral medications, and the vast majority of circulating influenza viruses have been susceptible to these medications. Among the influenza A (H3N2) viruses, 1,762 were tested for oseltamivir or zanamivir resistance and 1,128 were tested for peramivir resistance, and none were resistant. Among 32 influenza A (H1N1)pdm09 viruses tested for resistance to oseltamivir or peramivir, one (3%) was found to be resistant, and of the 28 viruses tested for resistance to zanamivir, none were found to be resistant. None of the 217 influenza B viruses tested were resistant to oseltamivir, zanamivir, or peramivir. High levels of resistance to the adamantanes (amantadine and rimantadine) persist among influenza A (H1N1)pdm09 and influenza A (H3N2) viruses.

## Outpatient Illness Surveillance

**FIGURE 2:**
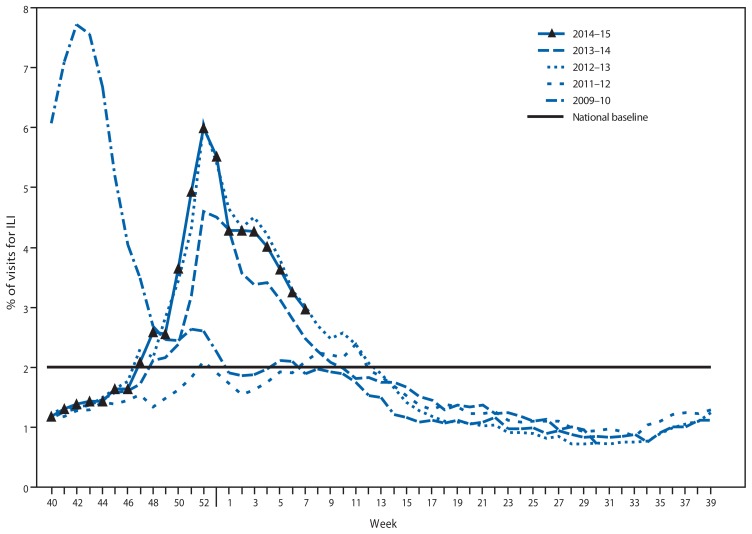
Percentage of visits for influenza-like illness (ILI)^*^ reported to CDC, by surveillance week — Outpatient Influenza-Like Illness Surveillance Network, United States, 2014–15 influenza season and selected previous influenza seasons ^*^ Defined as a fever (≥100°F [≥37.8°C]), oral or equivalent, and cough and/or sore throat, without a known cause other than influenza.

Since September 28, 2014, the weekly percentage of outpatient visits for influenza-like illness (ILI)§ reported by approximately 1,800 U.S. Outpatient ILI Surveillance Network (ILINet) providers in 50 states, New York City, Chicago, the U.S. Virgin Islands, Puerto Rico, and the District of Columbia that comprise ILINet, has ranged from 1.2% to 6.0%. From the week ending November 22, 2014 (week 47) to February 21, 2015 (week 7), the percentage equaled or exceeded the national baseline¶ of 2.0% for 14 consecutive weeks (Figure 2). During the 2001–02 through 2013–14 seasons, peak weekly percentages of outpatient visits for ILI ranged from 2.4% to 7.7% and remained above baseline levels for an average of 13 weeks (range = 1–19 weeks). For the week ending February 21, 2015 (week 7), all 10 U.S. Department of Health and Human Services regions** continued to report ILI activity at or above region-specific baseline levels.

Data collected in ILINet are used to produce a measure of ILI activity†† by jurisdiction. During the week ending February 21, 2015 (week 7), 11 states and Puerto Rico experienced high ILI activity (Arkansas, Connecticut, Kansas, Louisiana, Mississippi, New Jersey, New York, North Carolina, Oklahoma, Texas, and West Virginia), three states experienced moderate ILI activity (Colorado, Idaho, and Nevada), 16 states experienced low ILI activity (Alabama, California, Georgia, Hawaii, Massachusetts, Minnesota, Missouri, Pennsylvania, Rhode Island, South Carolina, South Dakota, Tennessee, Utah, Vermont, Virginia, and Wyoming), and 20 states and New York City experienced minimal ILI activity (Alaska, Arizona, Delaware, Florida, Illinois, Indiana, Iowa, Kentucky, Maine, Maryland, Michigan, Montana, Nebraska, New Hampshire, New Mexico, North Dakota, Ohio, Oregon, Washington, and Wisconsin). As of February 21, 2015, the largest total number of jurisdictions experiencing high ILI activity in a single week occurred during the weeks ending December 27, 2014 (week 52) and January 24, 2015 (week 3), when a total of 31 states and Puerto Rico experienced high ILI activity. A total of 45 jurisdictions have experienced high ILI activity at least 1 week this season. The peak number of jurisdictions experiencing high ILI activity in a single week during the last five influenza seasons has ranged from four during the 2011–12 season to 44 during the 2009–10 season.

## Geographic Spread of Influenza

For the week ending February 21, 2015 (week 7), the geographic spread of influenza§§ was reported as widespread in Guam and 20 states (Alabama, California, Connecticut, Delaware, Idaho, Indiana, Iowa, Maine, Maryland, Massachusetts, Mississippi, Montana, New Hampshire, New Jersey, New York, North Carolina, Oklahoma, Rhode Island, Vermont, and Virginia), regional in Puerto Rico, the U.S. Virgin Islands, and 25 states (Arizona, Arkansas, Florida, Georgia, Hawaii, Kansas, Kentucky, Louisiana, Michigan, Missouri, Nebraska, Nevada, New Mexico, North Dakota, Ohio, Oregon, Pennsylvania, South Carolina, Tennessee, Texas, Utah, Washington, West Virginia, Wisconsin, and Wyoming), and local in the District of Columbia and five states (Alaska, Colorado, Illinois, Minnesota, and South Dakota). As of February 21, 2015, the number of jurisdictions reporting influenza activity as widespread peaked during the weeks ending January 3, 2015 (week 53) and January 10, 2015 (week 1), when a total of 47 jurisdictions reported influenza activity as widespread. During the previous five seasons, the peak number of jurisdictions reporting widespread activity has ranged from 20 in the 2011–12 season to 49 in the 2010–11 season.

## Influenza-Associated Hospitalizations

**FIGURE 3:**
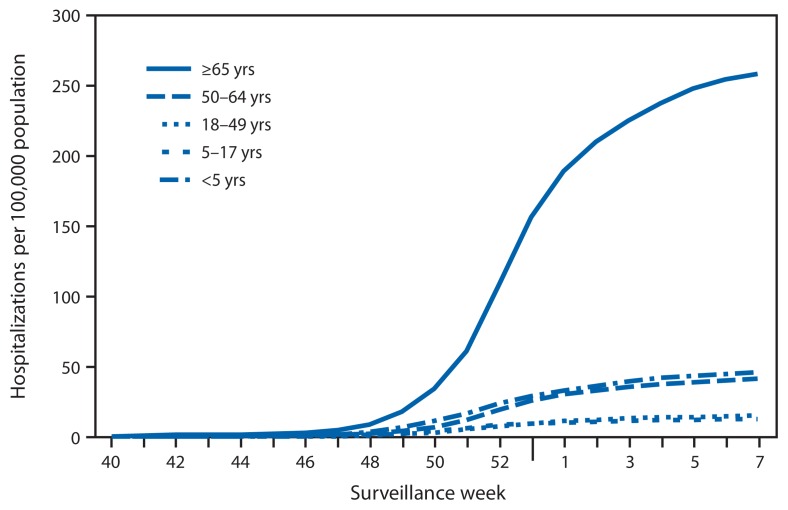
Cumulative rates of hospitalization for laboratory-confirmed influenza, by age group and surveillance week — FluSurv-NET,^*^ 2014–15 influenza season^†^ ^*^ FluSurv-NET conducts population-based surveillance for laboratory-confirmed influenza-associated hospitalizations among children aged <18 years (since the 2003–04 influenza season) and adults aged ≥18 years (since the 2005–06 influenza season). FluSurv-NET covers approximately 70 counties in the 10 Emerging Infections Program states (California, Colorado, Connecticut, Georgia, Maryland, Minnesota, New Mexico, New York, Oregon, and Tennessee) and additional Influenza Hospitalization Surveillance Project states (Michigan, Ohio, and Utah). ^†^ Data as of February 21, 2015.

CDC monitors hospitalizations associated with laboratory-confirmed influenza infection in adults and children through the Influenza Hospitalization Surveillance Network (FluSurv-NET),¶¶ which covers approximately 9% of the U.S. population. From October 1, 2014, through February 21, 2015, a total of 14,162 laboratory-confirmed influenza-associated hospitalizations were reported, with a cumulative rate thus far for all age groups of 51.7 per 100,000 population. The most affected age group was adults aged ≥65 years, accounting for more than 60% of reported influenza-associated hospitalizations. The cumulative hospitalization rate (per 100,000 population) from October 1, 2014, through February 21, 2015, was 45.7 among children aged <5 years, 12.9 among children aged 5–17 years, 15.0 among adults aged 18–49 years, 41.2 among adults aged 50–64 years, and 258.0 among adults aged ≥65 years (Figure 3). During the past three influenza seasons (2011–12 through 2013–14), end-of-season overall cumulative hospitalization rates ranged from 8.7 to 43.9 per 100,000 population, and age-specific cumulative hospitalization rates ranged from 16.0 to 67.0 per 100,000 population for ages <5 years, 4.0 to 14.6 for ages 5–17 years, 4.2 to 21.5 for ages 18–49 years, 8.1 to 53.7 for ages 50–64 years, and 30.2 to 183.2 for ages ≥65 years. Among all hospitalizations reported during the 2014–15 influenza season, 13,416 (94.8%) were associated with influenza A, 625 (4.4%) with influenza B, 46 (0.3%) with influenza A and B coinfection, and 67 (0.5%) had no virus type information. Among those with influenza A virus subtype information, 4,000 (99.7%) were A (H3N2) and 10 (0.2%) were A (H1N1)pdm09.

As of February, 21, 2015, and based on 3,118 (22.0%) cases with complete medical chart abstraction, the most commonly reported underlying medical conditions among hospitalized adults were cardiovascular disease, metabolic disorders, and obesity. The most commonly reported underlying medical conditions in hospitalized children were asthma, neurologic disorders, and immune suppression. Seven percent of adults and 39% of hospitalized children had no identified underlying medical conditions that placed them at higher risk for influenza complications.*** Among 253 hospitalized women of childbearing age (15–44 years), 67 (26%) were pregnant.

## Pneumonia and Influenza–Associated Mortality

**FIGURE 4:**
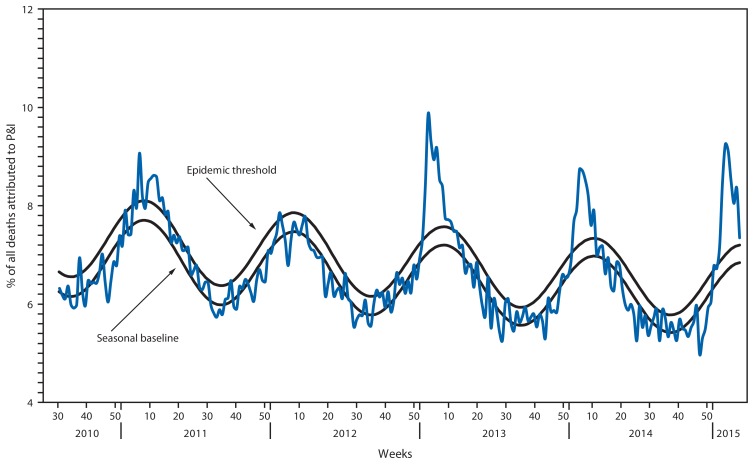
Percentage of all deaths attributable to pneumonia and influenza (P&I), by surveillance week and year^*^ — 122 Cities Mortality Reporting System, United States, 2010–2015 ^*^ Data as of February 21, 2015.

For the week ending February 21, 2015 (week 7), pneumonia and influenza (P&I) was reported as an underlying or contributing cause of death for 7.4% of all deaths reported to the 122 Cities Mortality Reporting System (Figure 4). This percentage is above the epidemic threshold of 7.2% for that week.††† Since September 28, 2014, the weekly percentage of deaths attributed to P&I ranged from 5.0% to 9.3%, and as of February 21, 2015 (week 7), had exceeded the epidemic threshold for 8 consecutive weeks (weeks ending January 3–February 21, 2015 [weeks 53–7]). The peak weekly percentages of deaths attributed to P&I for the previous five seasons ranged from 7.9% during the 2011–12 season to 9.9% during the 2012–13 season.

## Influenza-Associated Pediatric Mortality

As of February 21, 2015, a total of 92 laboratory-confirmed influenza-associated pediatric deaths that occurred during the 2014–15 season were reported to CDC from New York City and 31 states. The mean and median ages of children reported to have died were 7.2 and 5.9 years, respectively; 10 children were aged <6 months, 15 were aged 6–23 months, 14 were aged 2–4 years, 30 were aged 5–11 years, and 23 were aged 12–17 years. Of the 92 deaths, 43 were associated with an influenza A (H3N2) virus infection, 40 deaths were associated with an influenza A virus infection that was not subtyped, six deaths were associated with an influenza B infection, two deaths were associated with an influenza A and B coinfection, and one death was associated with an influenza virus for which the type was not determined. Since influenza-associated pediatric mortality became a nationally notifiable disease in 2004, the total number of influenza-associated pediatric deaths has ranged from 37 to 171 per season; excluding the 2009 pandemic, when 358 pediatric deaths were reported to CDC during April 15, 2009, through October 2, 2010.

### Discussion

The 2014–15 influenza season began early and, as of February 21, 2015, activity remained elevated across the United States. Influenza A (H3N2) viruses have been predominant overall, though in recent weeks an increasing proportion of influenza B viruses have been detected. Influenza A (H1N1)pdm09 viruses have been reported only rarely. Previous seasons during which influenza A (H3N2) viruses have predominated have often been associated with increased hospitalizations and deaths, especially among children aged <5 years and adults aged ≥65 years (3–5). The most recent previous season during which influenza A (H3N2) viruses predominated was in 2012–13. Although the current season has exhibited similar timing, data to date suggest it is more severe than the 2012–13 season for adults aged ≥65 years. The percentage of outpatient visits for ILI first exceeded the national baseline in mid-November (week 47) and, as of February 21, 2015, had remained above baseline for 14 consecutive weeks with a peak during late December. During the 2012–13 influenza season, similar ILI patterns were observed: the percentage of outpatient visits for ILI remained at or above baseline for 17 consecutive weeks, suggesting that influenza activity in the United States could continue this season for several more weeks. The highest rates of influenza-associated hospitalizations are generally observed among adults aged ≥65 years and children aged <5 years, and during seasons when influenza A (H3N2) viruses have predominated, higher hospitalization rates and mortality have been observed among these groups (3,6). This season, the highest rates of hospitalization have been among adults aged ≥65 years and are five-fold or greater than the overall and other age group-specific hospitalization rates. Through February 21, 2015, the cumulative rate of influenza-associated hospitalizations among this age group was 258.0 per 100,000 population, exceeding the cumulative total of 183.2 per 100,000 population for the entire 2012–13 season, which had been the highest previous recorded laboratory-confirmed influenza-associated hospitalization rate since this type of surveillance began in 2005. Among children aged <5 years, the cumulative hospitalization rate through February 21, 2015 (week 7) (45.7 per 100,000 population) is slightly less than that observed during the same week of the 2012–13 season (51.9 per 100,000 population). As of February 21, 2015, approximately 79% of the P&I deaths this season have occurred in adults aged ≥65 years and is similar to what was observed during the 2012–13 influenza season. However, the peak weekly percentage of deaths attributed to P&I for the current influenza season (9.3%) did not exceed the peak observed during the 2012–13 influenza season (9.9%).

A notable characteristic of the 2014–15 influenza season is that antigenic and genetic characterization of influenza-positive respiratory specimens submitted to CDC indicate that most of the circulating influenza A (H3N2) viruses are antigenically or genetically drifted from the influenza A (H3N2) component of the 2014–15 Northern Hemisphere vaccines (A/Texas/50/2012). Among the drifted viruses, most were antigenically similar to the influenza A (H3N2) virus selected for the 2015 Southern Hemisphere influenza vaccine (A/Switzerland/9715293/2013). A/Switzerland-like H3N2 viruses were first detected in the United States in small numbers in March 2014 and began to increase from July to September 2014 (1).

The predominance of drifted influenza A (H3N2) viruses has resulted in reduced vaccine effectiveness this season. Updated interim estimates of data collected from November 10, 2014 through January 30, 2015 indicate that overall the influenza vaccine was 19% (95% confidence interval (CI) = 7%–29%) effective in preventing medical visits across all age groups, and specifically, was 18% (CI = 6%–29%) and 45% (CI = 14%–65%) effective in preventing medical visits associated with influenza A (H3N2) and influenza B (Yamagata lineage), respectively (7). Although protection is reduced compared with previous seasons when most circulating and vaccine strain viruses were well-matched, influenza vaccination can still provide protection against vaccine-like influenza A (H3N2) viruses that have not undergone significant antigenic drift and influenza B viruses, which have predominated at the end of many influenza seasons (1,3,6). Although health care providers should continue to offer vaccine to all unvaccinated persons aged ≥6 months, antiviral medications are more important than usual as an adjunct to vaccination in the treatment and prevention of influenza. Recommended neuraminidase inhibitor antiviral medications include oseltamivir (Tamiflu), zanamivir (Relenza), and peramivir (Rapivab). Adamantane antiviral medications (rimantadine and amantadine) are not recommended because high levels of resistance persist among circulating influenza A viruses and they are not effective against influenza B viruses. Early treatment with antiviral medication can shorten the duration of influenza symptoms and reduce the risk for severe complications (8). A recent meta-analysis using individual patient data from published and unpublished randomized controlled clinical trials found that use of oseltamivir for the treatment of laboratory-confirmed influenza in adults reduced the time for symptoms to resolve by 21%, reduced the risk for lower respiratory tract complications by 44%, and reduced the risk for hospitalization by 63% compared with those treated with a placebo (9).

CDC recommends that antiviral treatment should be initiated as soon as possible after illness onset (ideally within 48 hours of symptom onset) for any patient with confirmed or suspected influenza who is hospitalized, has severe, complicated, or progressive illness, or is at high risk for influenza-associated complications, including children aged <2 years and adults aged ≥65 years. However, even when started after 48 hours of illness onset, antiviral treatment might still be beneficial in patients with severe, complicated, or progressive illness and in hospitalized patients. Antiviral treatment decisions should not be delayed awaiting laboratory confirmation of influenza (8).

Influenza surveillance reports for the United States are posted online weekly and are available at http://www.cdc.gov/flu/weekly. Additional information regarding influenza viruses, influenza surveillance, influenza vaccine, influenza antiviral medications, and novel influenza A infections in humans is available at http://www.cdc.gov/flu.

What is already known on this topic?

CDC collects, compiles, and analyzes data on influenza activity year-round in the United States. The timing and severity of circulating influenza viruses can vary by geographic location and season.

What is added by this report?

Influenza activity in the United States began to increase in mid-November, remained elevated through February 21, 2015, and is expected to continue for several more weeks. This has been an especially severe season for adults aged ≥65 years; this group has the highest recorded influenza-associated hospitalization rate and accounts for the majority of pneumonia and influenza–associated deaths this season. During September 28, 2014–February 21, 2015, influenza A (H3N2) viruses predominated. Characterization data indicate that most of the influenza A (H3N2) viruses have antigenically or genetically drifted and are different from the influenza A (H3N2) component of the 2014–15 Northern Hemisphere vaccines. The vast majority of currently circulating influenza viruses are sensitive to oseltamivir, zanamivir, and peramivir.

What are the implications for public health practice?

Although vaccine effectiveness is reduced this season, influenza vaccination remains the most effective way to prevent influenza illness. Antiviral medications are more important than usual as an adjunct to vaccination in the treatment and prevention of influenza. Early antiviral treatment is recommended for patients with severe, complicated, or progressive influenza illness and those at higher risk for influenza complications, including adults aged ≥65 years.

## Footnotes

* The CDC influenza surveillance system collects five categories of information from eight data sources: 1) viral surveillance (U.S. World Health Organization collaborating laboratories, the National Respiratory and Enteric Virus Surveillance System, and novel influenza A virus case reporting); 2) outpatient illness surveillance (U.S. Outpatient Influenza-Like Illness Surveillance Network); 3) mortality (122 Cities Mortality Reporting System and influenza-associated pediatric mortality reports); 4) hospitalizations (FluSurv-NET, which includes the Emerging Infections Program and surveillance in three additional states); and 5) summary of the geographic spread of influenza (state and territorial epidemiologist reports).

† CDC routinely uses hemagglutination inhibition (HI) assays to antigenically characterize influenza viruses year-round to compare how similar currently circulating influenza viruses are to those included in the influenza vaccine, and to monitor for changes in circulating influenza viruses. However, a portion of recent influenza A (H3N2) viruses do not grow to sufficient hemagglutination titers for antigenic characterization by HI. For many of these viruses, CDC is also performing genetic characterization to infer antigenic properties.

§ Defined as a temperature ≥100°F (≥37.8°C), oral or equivalent, and cough and/or sore throat, without a known cause other than influenza.

¶ The national and regional baselines are the mean percentage of visits for ILI during non-influenza weeks for the previous three seasons plus two standard deviations. Non-influenza weeks are defined as periods of 2 or more consecutive weeks in which each week accounted for less than 2% of the season’s total number of specimens that tested positive for influenza. National and regional percentages of patient visits for ILI are weighted on the basis of state population. Use of the national baseline for regional data is not appropriate.

** The 10 regions include the following jurisdictions: Region 1: Connecticut, Maine, Massachusetts, New Hampshire, Rhode Island, and Vermont; Region 2: New Jersey, New York, Puerto Rico, and the U.S. Virgin Islands; Region 3: Delaware, District of Columbia, Maryland, Pennsylvania, Virginia, and West Virginia; Region 4: Alabama, Florida, Georgia, Kentucky, Mississippi, North Carolina, South Carolina, and Tennessee; Region 5: Illinois, Indiana, Michigan, Minnesota, Ohio, and Wisconsin; Region 6: Arkansas, Louisiana, New Mexico, Oklahoma, and Texas; Region 7: Iowa, Kansas, Missouri, and Nebraska; Region 8: Colorado, Montana, North Dakota, South Dakota, Utah, and Wyoming; Region 9: Arizona, California, Hawaii, Nevada, American Samoa, Commonwealth of the Northern Mariana Islands, Federated States of Micronesia, Guam, Marshall Islands, and Republic of Palau; Region 10: Alaska, Idaho, Oregon, and Washington.

†† Activity levels are based on the percentage of outpatient visits in a state attributed to ILI and are compared with the average percentage of ILI visits that occur during weeks with little influenza virus circulation. Activity levels range from minimal, which would correspond to ILI activity from outpatient clinics being below or only slightly above the average, to high, which would correspond to ILI activity from outpatient clinics being much higher than the average.

§§ Levels of activity are 1) no activity; 2) sporadic: isolated laboratory-confirmed influenza cases or a laboratory-confirmed outbreak in one institution, with no increase in activity; 3) local: increased ILI, or at least two institutional outbreaks (ILI or laboratory-confirmed influenza) in one region of the state, with recent laboratory evidence of influenza in that region; virus activity no greater than sporadic in other regions; 4) regional: increased ILI activity or institutional outbreaks (ILI or laboratory-confirmed influenza) in at least two but less than half of the regions in the state with recent laboratory evidence of influenza in those regions; and 5) widespread: increased ILI activity or institutional outbreaks (ILI or laboratory-confirmed influenza) in at least half the regions in the state, with recent laboratory evidence of influenza in the state.

¶¶ FluSurv-NET conducts population-based surveillance for laboratory-confirmed influenza-associated hospitalizations in children aged <18 years (since the 2003–04 influenza season) and adults aged ≥18 years (since the 2005–06 influenza season). FluSurv-NET covers approximately 70 counties in the 10 Emerging Infections Program states (California, Colorado, Connecticut, Georgia, Maryland, Minnesota, New Mexico, New York, Oregon, and Tennessee) and additional Influenza Hospitalization Surveillance Project (IHSP) states. IHSP began during the 2009–10 season to enhance surveillance during the 2009 H1N1 pandemic. IHSP sites included Iowa, Idaho, Michigan, Oklahoma, and South Dakota during the 2009–10 season; Idaho, Michigan, Ohio, Oklahoma, Rhode Island, and Utah during the 2010–11 season; Michigan, Ohio, Rhode Island, and Utah during the 2011–12 season; Iowa, Michigan, Ohio, Rhode Island, and Utah during the 2012–13 season; and Michigan, Ohio, and Utah during the 2013–14 and 2014–15 seasons. Incidence rates are calculated using CDC’s National Center for Health Statistics population estimates for the counties included in the surveillance catchment area. Laboratory confirmation is dependent on clinician-ordered influenza testing, and testing for influenza often is underutilized because of the poor reliability of rapid test results and greater reliance on clinical diagnosis for influenza. As a consequence, cases identified as part of influenza hospitalization surveillance likely are an underestimation of the actual number of persons hospitalized with influenza.

*** Persons at higher risk include children aged <5 years (especially those aged <2 years); adults aged ≥65 years; persons with chronic pulmonary (including asthma), cardiovascular (except hypertension alone), renal, hepatic, hematologic (including sickle cell disease), metabolic disorders (including diabetes mellitus), or neurologic and neurodevelopment conditions (including disorders of the brain, spinal cord, peripheral nerve, and muscle, such as cerebral palsy, epilepsy [seizure disorders], stroke, intellectual disability [mental retardation], moderate to severe developmental delay, muscular dystrophy, or spinal cord injury); persons with immunosuppression, including that caused by medications or by human immunodeficiency virus infection; women who are pregnant or postpartum (within 2 weeks after delivery); persons aged ≤18 years who are receiving long-term aspirin therapy; American Indians/Alaska Natives; persons who are morbidly obese (i.e., body mass index ≥40); and residents of nursing homes and other chronic care facilities.

††† The seasonal baseline proportion of P&I deaths is projected using a robust regression procedure in which a periodic regression model is applied to the observed percentage of deaths from P&I that were reported by the 122 Cities Mortality Reporting System during the preceding 5 years. The epidemic threshold is set at 1.645 standard deviations above the seasonal baseline.
